# Proteomic Analysis of Signaling Network Regulation in Renal Cell Carcinomas with Differential Hypoxia-Inducible Factor-2α Expression

**DOI:** 10.1371/journal.pone.0071654

**Published:** 2013-08-05

**Authors:** Lokesh Dalasanur Nagaprashantha, Tatjana Talamantes, Jyotsana Singhal, Jia Guo, Rit Vatsyayan, Navin Rauniyar, Sanjay Awasthi, Sharad S. Singhal, Laszlo Prokai

**Affiliations:** Department of Molecular Biology and Immunology, University of North Texas Health Science Center, Fort Worth, Texas, United States of America; Moffitt Cancer Center, United States of America

## Abstract

**Background:**

The loss of von Hippel–Lindau (*VHL*) protein function leads to highly vascular renal tumors characterized by an aggressive course of disease and refractoriness to chemotherapy and radiotherapy. Loss of *VHL* in renal tumors also differs from tumors of other organs in that the oncogenic cascade is mediated by an increase in the levels of hypoxia-inducible factor-2α (HIF2α) instead of hypoxia-inducible factor-1α (HIF1α).

**Methods and Principal Findings:**

We used renal carcinoma cell lines that recapitulate the differences between mutant *VHL* and wild-type *VHL* genotypes. Utilizing a method relying on extracted peptide intensities as a label-free approach for quantitation by liquid chromatography–mass spectrometry, our proteomics study revealed regulation of key proteins important for cancer cell survival, proliferation and stress-resistance, and implicated differential regulation of signaling networks in *VHL*-mutant renal cell carcinoma. We also observed upregulation of cellular energy pathway enzymes and the stress-responsive mitochondrial 60-kDa heat shock protein. Finding reliance on glutaminolysis in *VHL*-mutant renal cell carcinoma was of particular significance, given the generally predominant dependence of tumors on glycolysis. The data have been deposited to the ProteomeXchange with identifier PXD000335.

**Conclusions and Significance:**

Pathway analyses provided corroborative evidence for differential regulation of molecular and cellular functions influencing cancer energetics, metabolism and cell proliferation in renal cell carcinoma with distinct *VHL* genotype. Collectively, the differentially regulated proteome characterized by this study can potentially guide translational research specifically aimed at effective clinical interventions for advanced *VHL*-mutant, HIF2α-over-expressing tumors.

## Introduction

Loss of the tumor suppressor von Hippel-Lindau (*VHL*) gene is an established genetic risk factor for renal cell carcinoma (RCC) and the affected individuals are at higher risk to develop tumors including pheochromocytomas, retinal angioma, pancreatic cysts and central nervous system hemangioblastomas [1], [2]. RCC is one of the top ten cancers incident in USA where *VHL*-gene alternations contribute to 75% of clear cell subtype of renal tumors, a major form of RCC [3].

Loss-of-function mutations in the *VHL* gene upregulate hypoxia-inducible factor-1α (HIF1α) and hypoxia-inducible factor-2α (HIF2α), but advanced stages of RCC have predominantly increased expression of HIF2α [4], [5]. Overexpression of HIF2α in RCC leads to enhanced angiogenesis and tumor progression even in the absence of HIF1α [6], [7]. In this regard, proteomic analyses have focused on molecular binding partners of *VHL* and interactions of *VHL*-related binding proteins [8], [9]. However, surveys have not been performed to reveal the specific nature of tumor cell processes and signaling networks that accompany the transformation to the *VHL*-mutant, HIF2α over-expressing (*VHL*-mut) RCC, particularly in relation to the *VHL* wild-type RCC lacking HIF2α (*VHL*-wt). Since 786-O cells express HIF2α, they represent a molecular environment of a *VHL*-mut genotype that corresponds to advanced stages of RCC [4], [10]. The drug sensitivity assays in our previously published reports have indicated an enhanced sensitivity of *VHL*-wt Caki-2 RCC cells to sorafenib compared to *VHL*-mut cells [11]. In addition, our preliminary proteomic analysis indicated enhanced expression of aldo-keto reductase family 1, member C1 (AKR1C1), and lack of expression of glutathione S-transferase (GST) in *VHL*-wt RCC [12]. Identification of further up- and downregulated proteins by specific *VHL* genotypes of RCC would enhance the understanding of the major regulators and help to streamline development of therapeutic strategies. Specifically, we aimed at comparing the global cellular proteome in 786-O (*VHL*-mut, over-expressing HIF2α) and Caki-2 (*VHL*-wt, lacking HIF2α) cell lines thereby focusing on HIF2α-dependence of RCC in this study.

Many laboratories take advantage of gel-free, bottom-up proteomics based on liquid chromatography (LC) coupled with mass spectrometry (MS) and tandem mass spectrometry (MS/MS) [13]–[16]. Previously, we have reported a comparative analysis of data-dependent shotgun LC-MS/MS with 2D-LC-MS/MS [17]. Results demonstrated regulation of relevant biological processes using either method further indicating suitability of a shotgun approach for initial, “screening-driven” proteomic applications [17] with a label-free strategy to afford relative protein expression differences based on spectral counts [18]. In this proteomics study, we performed proteomic analysis of *VHL*-wt and *VHL*-mut RCC cells using peptide intensities extracted from full-scan MS spectra to enable label-free quantification that overcomes limitations of the spectral counting method [19]. This improved technique enabled the identification of critical protein expression differences regulating metabolism, oxidative stress, and proliferation according to the *VHL*-genotype of RCC.

## Materials and Methods

### Chemicals

All chemicals and reagents were purchased from Sigma Aldrich (St. Louis, MO) unless otherwise specified.

### Cell Lines and Cultures

No permits were required for the described study, which complied with all relevant regulations. The Caki-2 *VHL*-wt human RCC cell line (HTB-47™), established from renal carcinoma of a male Caucasian patient, was purchased from the American Type Culture Collection (Manassas, VA). The 768-O *VHL*-mut cell line (CRL-1932™), also available from the American Type Culture Collection and derived from renal carcinoma of another male Caucasian patient, were kindly authenticated and provided by Dr. William G. Kaelin, Dana-Farber Cancer Institute and Harvard Medical School (Boston, MA). These *VHL*-mut (786-O) and *VHL*-wt (Caki-2) cell lines are, thus, normalized for histologic origin (epithelial *versus* mesenchymal part of kidney), sex (male) and race (both of Caucasian origin). All cells were cultured at 37°C in a humidified atmosphere of 5% CO_2_ in RPMI-1640 medium supplemented with 10% FBS and 1% P/S solution. Before proteomic analysis, we confirmed the differential expression of HIF2α between the RCC cell lines (**Fig. S1**).

### Cell Viability Assay

Cell density measurements were done using a hemocytometer to count reproductive cells resistant to staining with trypan blue. Approximately 20,000 cells were plated into each well of 96-well flat-bottomed micro-titer plates. After a 12-h incubation at 37°C, RPMI-1640 medium containing different concentrations of aminooxyacetate (AOA, ranging from 0.1 to 5 mM) were added to the cells. After 24, 48 and 72 h of incubation, respectively, 20 µl of 5 mg/ml 3-(4,5-dimethylthiazol-2-yl)-2,5-diphenyltetrazolium bromide (MTT) were introduced to each well and incubated for 2 h of exposure. The plates were centrifuged and medium was decanted. Cells were subsequently dissolved in 100 µl DMSO with gentle shaking for 2 h at room temperature, followed by measurement of optical density at 570 nm [11]. For assessment of cell viability in medium without glutamine supplementation, the cells were plated and after 12 h of incubation at 37°C in RPMI-1640 with glutamine the medium was changed to RPMI-1640 medium without glutamine. The MTT assay was performed at 24, 48, and 72 h, as indicated above. Four replicate wells were used at each point in each of the three separate measurements.

### Sample Preparation

RCC cells were resuspended in buffer containing 20 mM Tris-HCl, 50 mM NaCl, and 6 M urea, 10 mM sodium pyrophosphate, 1 mM sodium fluoride and 1 mM sodium orthovanadate and incubated on ice for 30 min followed by six freeze thaw cycles to ensure adequate lysis of the cells. The sample was centrifuged at 13,300 rpm for 30 min at 4°C. The clear supernatant was collected carefully and protein concentration was measured using BCA assay (Bio-Rad, CA). Each sample equivalent to 200 µg of protein was reduced using 2.5 mM dithiothreitol (DTT) at 65°C for 30 min followed by carbamidomethylation of thiol groups using 7 mM iodoacetamide for 30 min at room temperature in the dark. The unreacted iodoacetamide was quenched by addition of DTT to 2.5 mM with additional 15 min incubation. The sample was then diluted in 50 mM ammonium bicarbonate to lower the urea concentration to less than 2 mM followed by digestion with sequencing grade trypsin (Promega, Madison, WI) for 15 h at enzyme: substrate ratio of 1∶25. Following digestion, proteolytic activity was terminated by acidifying the reaction mixture with acetic acid to pH<3. The samples were desalted using C_18_ cartridges (Supelco, Bellefonte, PA) and, then, the eluate from the cartridge was evaporated to dryness by lyophilizing at 4°C. The residues were reconstituted in 25 µL of loading solvent containing 0.1% (v/v) acetic acid and 5% (v/v) acetonitrile in 94.9% (v/v) water. Five μL aliquots were used for LC–MS/MS analyses.

### Liquid Chromatography–Mass Spectrometry

LC–ESI-MS and MS/MS analysis of the samples was performed using a hybrid linear quadrupole ion trap–Fourier transform ion cyclotron resonance (7-T) mass spectrometer (LTQ-FT, Thermo Finnigan, San Jose, CA) equipped with the manufacturer's nano-electrospray ionization source and operated with Xcalibur (version 2.2) data acquisition software. Online reversed-phase HPLC (RP-HPLC) was carried out with an Eksigent nano-LC-2D (Eksigent, Dublin, CA) system. Aliquots of 5 µL of the sample was automatically loaded onto an IntegraFrit™ sample trap (2.5 cm×75 µm) (New Objective, Woburn, MA) at a flow rate of 1.5 µl/min in a loading solvent containing 0.1% (v/v) acetic acid and 5% (v/v) acetonitrile in 94.9% (v/v) water prior to injection onto a reversed-phase column (NAN75-15-03-C18-PM; 75 µm i.d.×15 cm, LC Packings, Sunnyvale, CA) packed with C_18_ particles (3 µm, 100 Å pore size, PepMap). Mobile-phase solvent A consisted of 0.1% (v/v) acetic acid and 99.9% (v/v) water, and mobile-phase solvent B consisted of 0.1% (v/v) acetic acid and 99.9% (v/v) acetonitrile. Following desalting and elution from the sample trap onto the analytical column, peptides were separated using the following gradient conditions: (1) 5 min in 95% solvent A for equilibration; (2) linear gradient to 40% solvent B over 90 min and holding at 40% solvent B for isocratic elution for 5 min; (3) increasing the gradient to 90% solvent B and maintaining for 5 min; and finally (4) 95% solvent A in the next 20 min. The flow rate was maintained at 250 nL/min. Peptides eluted through a Picotip emitter (internal diameter 10±1 µm; New Objective) were electrosprayed into the IonMax interface of the mass spectrometer. Spray voltage and capillary temperature were maintained at 2.0 kV and 250°C, respectively, during the gradient run. Using the automatic gain control mode of trapping in the LTQ, data-dependent mode of acquisition was utilized in which a survey scan was performed by Fourier-transform ion cyclotron resonance (FTICR) mass spectrometry to select the top five most intense precursor ions for parallel MS/MS linear ion trap (LTQ) analysis, while FTICR full-scan mass spectra were acquired at 50000 mass resolving power (*m/z* 400) from *m/z* 350 to 1500. Peptide fragmentation was performed by collision-induced dissociation (CID) using a 3.0-Th isolation width and 35% normalized collision energy with helium as the collision gas. The precursor ion that had been selected for CID was dynamically excluded from further MS/MS analysis for 60 s.

### Database Search

MS/MS data generated by data dependent acquisition via the LTQ-FT were extracted by BioWorks version 3.3 and searched against a composite IPI human protein sequence database (IPI _human_v 3.73.par) containing both forward and randomized sequences using Mascot version 2.2 (Matrix Science, Boston, MA) search algorithm. Mascot was searched with a fragment ion mass tolerance of 0.80 Da and a parent ion tolerance of 15.0 ppm assuming trypsin as the digestion enzyme with the possibility of one missed cleavage. Carbamidomethylation of cysteine was specified as a fixed modification, while oxidation of methionine, N-terminal protein acetylation, N-terminal peptide and lysine carbamoylation, and phosphorylation of serine and threonine were specified as variable modifications.

### Data Compilation, Relative Quantification and Analysis of Signaling Networks

The analysis of *VHL*-wt (Caki-2) human RCC and *VHL*-mut (786-O) cells was carried out from three independent, biological samples from each cell line (tryptic digests of Caki-2 and 786-O, respectively) and duplicate injections were made for each sample. Data processing was accomplished using two software tools: Scaffold (version Scaffold 3.0, Proteome Software Inc., Portland, OR) and Progenesis LC-MS (version 3.0, Nonlinear Dynamics, Durham, NC). Scaffold was employed to validate MS/MS-based peptide and protein identifications from Mascot database searching. Initial peptide identifications were accepted if they could be established at greater than 95% probability as specified by the Peptide Prophet algorithm [20]. Protein probabilities were assigned by the Protein Prophet algorithm [21]. Protein identifications were accepted, if they reached greater than 99% probability and contained at least 2 identified unique peptides. These identification criteria typically established <0.01% false discovery rate based on a decoy database search strategy at the protein level.

Extracted peptide intensities were evaluated with Progenesis LC-MS to obtain label-free relative quantification from the raw data files. The software created a single aggregate run based on LC retention time of each analysis to contain all MS data with distinguished peptide ions and, then, further feature outline maps were generated for detection and quantification of peptide ions from individual analyses using default program parameters. Peptide quantifications were performed by summing peak intensities followed by normalization, data filtering to retain only ions with positive charges (*z*) of two and three, and exporting peak lists to query against the IPI human protein sequence database using Mascot (see *Database Search* subsection above). Proteins were accepted as differentially expressed following analysis of variance (ANOVA, *P*<0.05), requiring at least a twofold change in expression and also passing validation by Peptide Prophet and Protein Prophet using protein identifications from Scaffold. The mass spectrometry proteomics data have been deposited to the ProteomeXchange Consortium (http://proteomecentral.proteomexchange.org) via the PRIDE partner repository [22] with the dataset identifier PXD000335 and DOI 10.6019/PXD000335.

### Statistical Analyses

Considering data exported from Progenesis LC-MS, samples from each given subject appear in only one condition (i.e., *VHL*-wt *versus VHL*-mut). The ANOVA calculation assumed that the conditions were independent, and applied the statistical test for the hypothesis that the means were equal. Data values with *P*<0.05 were considered statistically significant. Differentially expressed proteins meeting criteria for statistical significance (*P*<0.05) by Progenesis LC-MS were further explored by Ingenuity Pathways Analysis (IPA; Ingenuity Systems, Redwood City, CA) to reveal differentially regulated signaling networks and biological processes.

### Western-blot Analyses

The RCC cells were lysed and analyzed by Western-blot for studying the expression of mitochondrial 60-kDa heat shock protein (HSPD1) and HIF-2α by using specific antibodies. Briefly, lysates containing ∼50 µg of proteins were subjected to sodium dodecyl sulfate–polyacrylamide gel electrophoresis (SDS-PAGE) and proteins were transferred onto nitrocellulose membrane. After blocking with 5% non-fat dry milk, the membrane was incubated overnight with the desired primary antibody (1∶1000 dilution). Subsequently, the membrane was incubated with appropriate secondary antibody for 2 h, and the immune-reactive bands were visualized using the enhanced chemiluminescence kit from Perkin-Elmer (Waltham, MA) according to the manufacturer's instructions. The same membrane was reprobed with the antibody against GAPDH (1∶5000 dilution) as an internal control for equal protein loading. The intensity of immune-reactive bands on Western blots was determined using a densitometer (Molecular Dynamics, Sunnyvale, CA) equipped with Image QuaNT software.

## Results and Discussion

The 786-O and Caki-2 RCC cell lines, which differ in HIF 2α, recapitulate the differences between *VHL*-wt and *VHL*-mut RCC. Furthermore, they have been extensively used for RCC studies [6], [23], [24]. Before proteomic analysis, we confirmed the differential expression of HIF-2α between the RCC cell lines (**Fig. S1**).

Our method of choice for this first study in RCC varying in HIF-2α expression was based on our earlier comparison of 2D-LC-MS/MS results with those obtained from shotgun LC-MS/MS analyses demonstrating that most molecular functions and biological processes were represented via gene ontology (GO) analysis using either methodology [17]. The analytical replications were >95% across all the samples analyzed in both *VHL*-wt and *VHL*-mut cells. We employed rigorous criteria for the evaluation of significant protein expression differences between *VHL* genotypes of RCC. Specifically, we required proteins to match Scaffold protein IDs, as well as have a minimum 2-fold change and an ANOVA *P*-value of <0.05 as determined by Progenesis LC-MS analyses (MS).

The introduction of Progenesis LC-MS into our workflow of label-free proteomics we employed earlier has resulted in a substantial improvement over previous software which was implemented for quantitative expression profiling based on spectral counting [18]. We have been able to verify a higher proportion of differentially regulated proteins obtained by integration of extracted peptide intensities (via Progenesis LC-MS software), which would have been excluded due to criteria applied to the evaluation of spectral counts (e.g., requiring minimum spectral counts and using stringent *G*-tests). Apparently, statistics applied to extracted peptide ion intensities did not compromise evaluation of lower-abundance proteins for differential expression, which would support arguments to phase out spectral counting methods in future quantitative studies [19]. **Table 1** lists representative *VHL* genotype- regulated RCC proteins. Additional proteins and peptides identified from Mascot database searching can be found in the Supporting Information (**Table S1**).

**Table 1:** Representative differentially expressed proteins of *VHL*-wt (Caki-2) and *VHL*-mut (786-O) RCCs identified by database search (Mascot) and quantified by extracted peptide intensity (MS) features generated with Progenesis LC-MS.

Accession	Protein Name^a^	Molecular Weight (kDa)	*P* ANOVA (*P*<0.05)	Max Fold Change	*VHL* mutant (786-O) Average Normalized Abundance ± Standard Deviation	*VHL*-wild type (Caki-2) Average Normalized Abundance ± Standard Deviation	Number of Unique Peptides	Sequence Coverage (%)
**A) Up-regulated in ** ***VHL*** **-mutant (786-O)**								
IPI00291006	Malate dehydrogenase, mitochondrial (*MDH2*)	36	0.006	7.5	6.35E+04±3.20E+04	8.51E+03±6.04E+03	11	23
IPI00303476	ATP synthase subunit beta, mitochondrial (*ATP5B*)	57	0.01	8.3	1.11E+05±6.35E+04	1.32E+04±6.73E+03	14	25
IPI00440493	ATP synthase subunit alpha, mitochondrial (*ATP5A1*)	60	0.01	42.2	1.80E+04±9.68E+03	4.27E+02±2.66E+02	7	12
IPI00784154	60 kDa heat shock protein, mitochondrial (*HSPD1*)^b^	61	0.005	13.2	5.79E+05±3.64E+05	4.40E+04±1.52E+04	23	40
**B) Down-regulated in ** ***VHL*** **-mutant (786-O)**								
IPI00027497	Glucose-6-phosphate isomerase (*GPI*)	63	0.03	3.7	1.37E+04±6.63E+03	5.12E+04±2.01E+04	7	12
IPI00031420	UDP-glucose 6-dehydrogenase (*UGDH*)	55	<0.001	10.7	1.98E+04±2.76E+03	2.12E+05±6.45E+04	18	41

a Gene symbols are given in parentheses.

b Validated by Western blot (**Fig. S3**).

**Figure 1:**
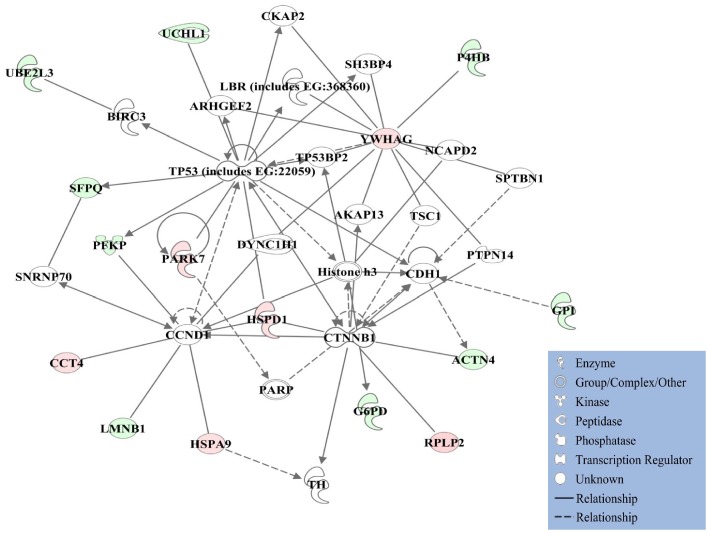
Ingenuity Pathway Analyses (IPA). Proteins with significant differential expression regulate a network of cell-to-cell signaling and interaction, cellular growth and proliferation, as well as cellular development. Red shading: upregulated in *VHL*-mut RCC; green shading: downregulated in *VHL*-mut RCC. Protein–protein interactions from the network diagram are represented by single lines and proteins/compounds that regulate another protein are indicated by arrows. Solid or dashed lines indicate direct or indirect interactions, respectively. The various shapes represent different protein functions.

**Figure 2:**
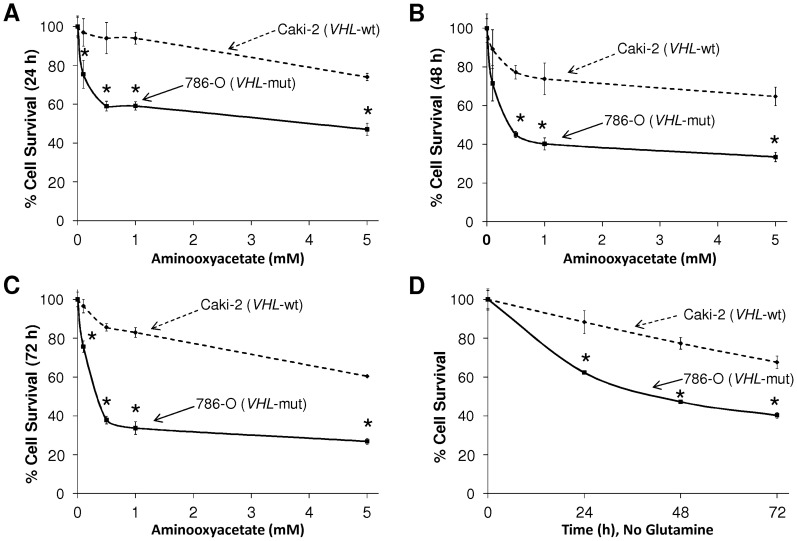
Assessment of cell-survival by MTT assay. The survival of Caki-2 (*VHL*-wt) and 786-O (*VHL*-mut) RCC cell lines was assessed after treatment with increasing concentrations of the glutaminolytic inhibitor, aminooxyacetate (AOA) at 24 h (**a**), 48 h (**b**) and 72 h (**c**) and in glutamine-depleted medium (**d**). *VHL*-mut 786-O RCC cells were more susceptible to inhibition of glutaminolysis or glutamine depletion at all time-points (* indicates statistically significant difference from *VHL*-wt control by the *t*-test, *P*<0.05, n = 4).

IPA generated four networks from differentially expressed proteins. Proteins with significant differences in expression were involved in regulation of cell-to-cell signaling and interaction, tissue development, and cancer (Network-1; **Fig. 1**). In this network, the well-known tumor suppressor gene TP53 takes an apparent central role. TP53 mutations are the most frequent genetic alterations found in human cancer [25], and renal pathology and DNA adduct analysis have identified the gene as one of the most important pathways modulated in the kidney by human carcinogens [26]. In addition, a network was identified for nucleic acid metabolism, small molecule biochemistry, cellular assembly and organization (Network-2, **Fig. S2A**) with presumed involvement of the Myc oncogene [27] under the direct control of the *VHL*-mut downregulated 40S ribosomal protein S20 (RSP20) and myosin-9 (MYH9), as well as through interaction with the *VHL*-mut upregulated heterogeneous nuclear ribonucleoprotein K (HNRNPK) according to our proteomic analysis of 786-O and Caki-2 RCC cell lines (**Table S1**). Networks associated with cellular development, cellular growth and proliferation, hematological system development and function (Network-3, **Fig. S2B**), hereditary disorder, metabolic disorder, and carbohydrate metabolism (Network-4, **Fig. S2C**) were also revealed by IPA. The complex network that could be built from combining Networks 1–4 was given in the Supporting Information (**Fig. S2D**). The quantitative difference in the expression of HSPD1, a protein found to be an important node in two networks (1 and 3, **Fig. 1** and **Fig. 2B**) was confirmed by Western blot analyses (**Fig. S3**).

### Differential Regulation of Cellular Energy Pathways in *VHL*-mut and *VHL*-wt RCC Cells

IPA revealed signaling pathways that regulate tumor energetics such as protein ubiquitination, methylglyoxal degradation III, glycolysis, gluconeogenesis, and pentose phosphate pathways in RCC cells according to *VHL*-genotype (**Table 2**). Differentially expressed proteins (**Table 1** and **Table S1**), along with their impact on the cellular energy metabolism as implicated by the IPA analysis (**Table 2**), also indicated significant differences in metabolic preferences of *VHL*-mut *versus VHL*-wt RCC. Metabolism in cancer cells is mainly driven by the flux of metabolites through three processes: glycolysis, pentose phosphate pathway and glutaminolysis [28]. Rapidly proliferating cancer cells have enhanced aerobic glycolysis compared to non-malignant cells, which is commonly known as Warburg effect [29]. Previously published proteomic studies have strongly indicated the up-regulation of glycolysis in RCC cells [30], [31]. However, pathways that maintain cellular energy status specifically in HIF2α over-expressing *VHL*-mut background, which corresponds to an advanced stage of RCC, have not been reported. The tumor cells with impaired glycolysis maintain the downstream metabolite flux by deriving the intermediate metabolites, fructose 6-phosphate and dihydroxyacetone phosphate, from the pentose phosphate pathway [28]. To our surprise, glucose-6-phosphate isomerase (GPI) of the glycolytic pathway (**Fig. 1** and **Table 1**) and UDP-glucose 6-dehydrogenase (UGDH) of the pentose phosphate pathway (**Fig. S2A** and **Table S1**) were downregulated. However, a significant upregulation of mitochondrial malate dehydrogenase 2 (MDH2) was observed in *VHL*-mut RCC cells (**Fig. S2B** and **Table S1**). The differential expression of cytosolic GPI of the glycolytic pathway and mitochondrial MDH2 of the glutaminolytic pathway were strongly supported by Progenesis LC-MS analysis based on extracted peptide intensities (**Fig. S4** and **Fig. S5**). This novel finding strongly indicated a possible metabolic shift towards glutaminolysis in advanced stages of RCC that overexpress HIF2α. Glutamine is converted to glutamate and shunted into the tricarboxylic acid (TCA) cycle as α-ketoglutarate that ultimately yields malate. MDH2 converts malate to oxaloacetate to afford NADH that is further shuttled into mitochondrial respiratory chain to yield ATP by the action of ATP synthase [32], [33]. In addition to mitochondrial MDH2, ATP synthase α and β (ATP5A1 and ATP5B; **Table 1** and **Fig. S2A**) also were overexpressed in *VHL*-mut RCC. Thus, the upregulation of mitochondrial MDH2, ATP5A1 and ATP5B in *VHL*-mut cells, in conjunction with downregulation of cytosolic GPI, strongly indicated a shift towards alternative glutaminolytic metabolic pathway in RCC associated with an advanced stage of malignancy.

**Table 2:** IPA Analysis: Cellular Pathways Significantly Represented by HIF2α Regulation in RCC Cell Lines.

*Top Pathways Differentially regulated in VHL-mut and VHL-wt RCC*	
Canonical Pathways	P-value
Glycolysis I	3.87E-05
Gluconeogenesis I	3.87E-05
Protein Ubiquitination Pathway	4.13E-05
Pentose Phosphate Pathway (Non-oxidative Branch)	8.99E-05
Methylglyoxal Degradation III	4.89E-04

In the context of downregulation of key glycolytic and pentose phosphate pathway enzymes, especially GPI and UGDH, revealed by proteomic analysis of *VHL*-mut RCC, we further investigated the importance of glutaminolysis. We first analyzed the degree of cell survival using MTT assays at 24, 48 and 72 h during incubation with aminooxyacetate (AOA), an inhibitor of glutaminolysis (**Fig. 2A**–**C**). The *VHL*-mut RCC cells were more susceptible to inhibition of glutaminolysis relative to *VHL*-wt RCC cells at all concentrations of AOA and at all time-points tested. In addition to using glutaminolytic inhibitor, we also studied the extent of cell survival upon culturing in medium lacking glutamine. Again, *VHL*-mut RCC cells were more susceptible to glutamine depletion than *VHL*-wt RCC cells (**Fig. 2D**).

By comparing the results of cell survival assays after glutaminolytic pathway inhibition by using AOA and glutamine depletion by using medium lacking glutamine, we observed that *VHL*-mut cells cultured in glutamine depleted medium had moderately higher survival when compared to survival upon treatment with the glutaminolytic inhibitor. This could be due to the presence of glutamate in the cell culture medium, which could compensate for glutamine deprivation when cultured without glutamine. By acting downstream of glutamate, the increased inhibitory effect of AOA is due to its ability to prevent conversion of glutamine to α-ketoglutarate, which would explain the low survival of *VHL*-mut RCC cells in the presence of AOA when compared to lack of glutamine substitution in the medium alone. Collectively, when compared with *VHL*-wt RCC, the cell survival assay both in the presence of AOA and upon cell cultures in glutamine-depleted medium confirmed the predominant dependence of *VHL*-mut RCC on active glutaminolytic pathway for survival.

### Regulation of Complex Cellular Energetics, Proliferative and Metastatic Signaling Networks in *VHL*-mut RCC

The differential regulation of various cellular processes, systemic protein networks, and toxicological pathways are increasingly becoming a point of reference in both enhancing the understanding about critical players in tumor biology as well as in designing prudent monotherapies and multi-drug combinations for aggressive cancers. In this regard, the IPA analyses revealed the pathways, potential interactions, and networks of differentially regulated proteins in advanced *VHL*-mut RCC, which can guide further interventional studies in RCC. The top metabolic cellular pathways that were differentially regulated included glycolysis, gluconeogenesis, protein ubiquitination pathway, and pentose phosphate pathway (**Table 2**). These dominant differences in energy metabolism pathways provide additional corroborative evidence for the significance of decreased glycolysis and increased glutaminolytic preferences of *VHL*-mut RCC *versus VHL*-wt RCC, which was also confirmed by additional molecular assays in this study. Given the increasing interventional focus on targeting specific metabolic transformation and dependencies in cancers, these differential metabolic pathways revealed by IPA could guide such integrated metabolic targeted therapies in RCC [34].

The top differentially regulated toxicological pathways were related to hypoxia-inducible factor signaling and oxidative stress (**Table 3**). Moderate levels of oxidative stress stimulate cancer cell proliferation, whereas high levels of ROS are toxic even to cancer cells [35]. The current drug of choice in RCC sorafenib not only inhibits tyrosine kinases, but also induces cell death through induction of mitochondrial oxidative stress [36]. Thus, the differential regulation of key networks of energy metabolism and oxidative stress pathways assumes significance in further directing the investigations into molecular mechanisms that regulate differential drug sensitivity for RCC drugs like sorafenib. In this context, the regulation of HSPD1 by the *VHL*-genotype of RCC may deserve attention. The heat shock family of proteins is one of the major regulators of the cellular response to oxidative stress and, hence, regulates the response to chemotherapeutic interventions [37]. HSPD1 is a predominant mitochondrial protein involved in maintaining the integrity of mitochondrial proteins and its expression is increased in response to ROS generation induced due to hypoxia, high temperatures and upon exposure to toxic chemicals [38].

**Table 3:** IPA Analysis: Toxicological Cellular Pathways Significantly Represented by HIF2α Regulation in RCC.

*Top Toxicological Pathways and Functions Differentially Associated with VHL-mut and VHL-wt RCC Cell Lines*		
Toxicological Pathways	P-value	
Mitochondrial Dysfunction	7.75E-04	
Hypoxia-Inducible Factor Signaling	1.24E-03	
Aryl Hydrocarbon Receptor Signaling	9.66E-03	
Oxidative Stress	1.25E-02	
Decreases Permeability Transition of Mitochondria and Mitochondrial Membrane	1.51E-02	

Name	P-value	Molecules^a^
*Nephrotoxicity*		
Glomerular Injury	8.04E-04 – 8.04E-04	ACTN4, MYH9
Renal Inflammation	6.08E-03 – 4.70E-02	ACTN4, MYH9
Renal Nephritis	6.08E-03 – 4.70E-02	ACTN4, MYH9
Nephrosis	3.30E-02 – 3.30E-02	ACTN4
Kidney Failure	1.36E-01 – 1.36E-01	MYH9
*Hepatotoxicity*		
Hepatocellular Carcinoma	6.96E-05 – 4.47E-02	AKR1B10, CTSD, HSPA5, NQO1, PFN1, TXN, UCHL1, YWHAG
Liver Hyperplasia/Hyperproliferation	6.96E-05 – 4.47E-02	AKR1B10, CTSD, HSPA5, NQO1, PFN1, TXN, UCHL1, YWHAG
Liver Cirrhosis	6.08E-03 – 2.81E-02	KRT18, HNRNPK, NQO1
Liver Cholestasis	1.65E-01 – 1.65E-01	AKR1C3
*Cardiotoxicity*		
Cardiac Damage	3.04E-03 – 3.04E-03	TXN
Cardiac Arteriopathy	5.24E-01 – 5.24E-01	TKT

a Symbol abbreviations are gene.

**Figure 3:**
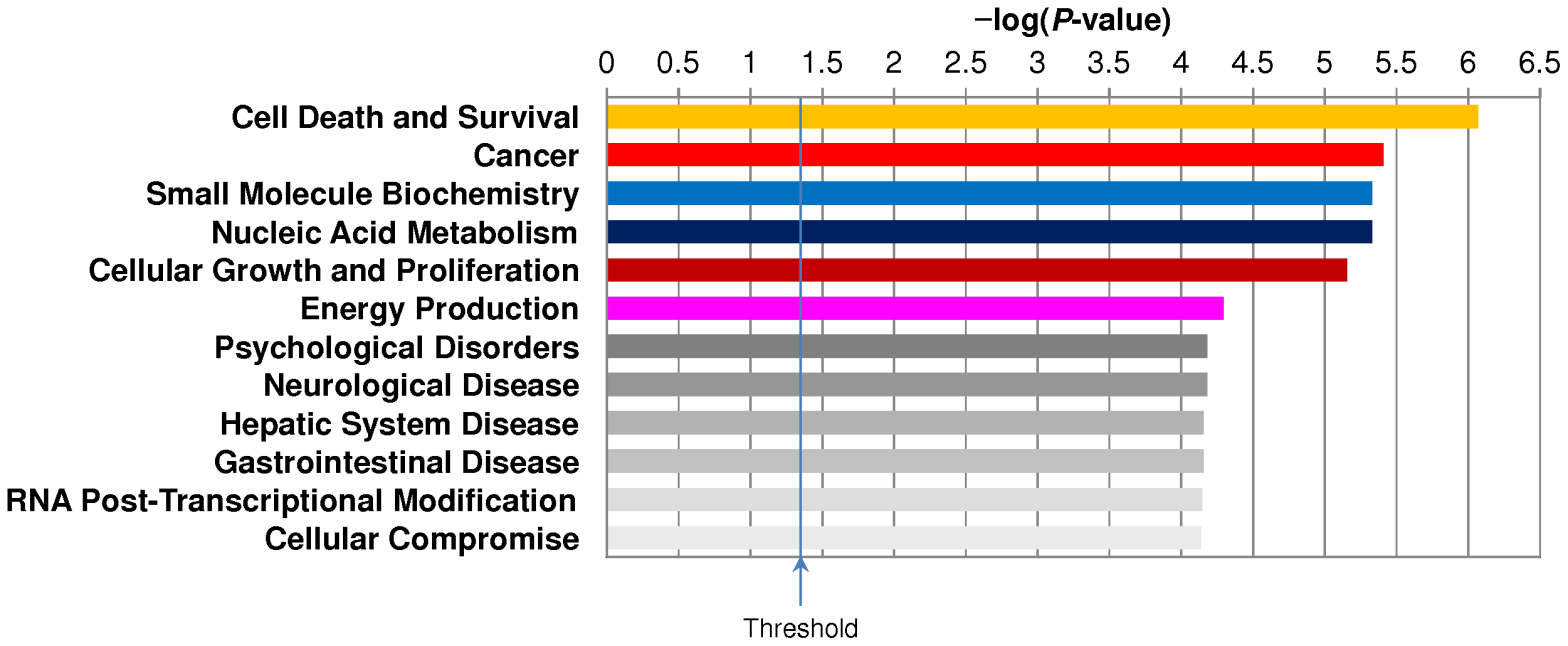
Summary of Ingenuity Pathways Analyses (IPA) revealing differentially regulated molecular and cellular functions in *VHL*-mut RCC compared to *VHL*-wt RCC.

Finally, the top differentially regulated molecular and cellular functions between *VHL* genotypes of RCC were cell death and survival, cancer, nucleic acid metabolism, cellular growth and proliferation, and energy production (**Fig 3**). Other significant differentially regulated cellular functions included RNA post-transcriptional modification and cellular compromise which are of fundamental relevance to cancer cell survival and proliferation.

## Conclusions

Proteomic studies focused on the tumorigenic and metastatic causative factors of particular importance in RCC specifically overexpressing HIF2α have potential impact on translational research to steer the course of novel therapeutic interventions. In this regard, we detected significant differential expression of proteins that regulate cancer cell energetics and oxidative stress.

First, this study on RCC differing in *VHL*-genotype and HIF2α levels identified quantitatively significant differential regulation of numerous proteins (**Table 1** and **Table S1**). Second, we also reported a novel finding of downregulation of glycolytic pathway and dependency of *VHL*-mut RCC on glutaminolysis, when compared to *VHL*-wt RCC (**Fig. 2**). In addition, IPA revealed a collective impact of differentially regulated proteins on vital pathways, processes, and networks (**Table 2**, **Table 3**, **Figures 1**, **S2A**–**D** and **3**). This has thereby provided a conceptual framework to facilitate further molecular and cellular biology studies, as well as to serve as a reference to design potential mono- and combinatorial therapies targeting these proteins and networks in advanced *VHL*-mut and HIF2α overexpressing RCC. In summary, results from this study may open potential novel possibilities for translational research in renal oncology by having characterized differentially regulated proteins and respective signaling networks between *VHL* genotypes of RCC differing specifically in HIF2α expression.

## Supporting Information

Figure S1 **Western-blot analysis for the expression of HIF2α in 786-O (** ***VHL*** **-mut) and Caki-2 (** ***VHL*** **-wt) RCC cell lines.**
(PDF)

Figure S2 **Ingenuity Pathway Analyses (IPA).** Proteins with significant differential expression regulate: **A.** A network of nucleic acid metabolism, small molecule biochemistry, cellular assembly and organization; **B.** A network of cellular development, cellular growth and proliferation, hematological system development and function; **C.** A network of hereditary disorder, metabolic disorder, carbohydrate metabolism; **D.** The complex, merged network that can be built from networks shown in **Fig. 1** and **Fig. S2A**–**C**. Red shading: up-regulated in *VHL*-mut RCC; green shading, down-regulated in *VHL*-mut RCC. Protein–protein interactions from the network diagram are represented by single lines and proteins/compounds that regulate another protein are indicated by arrows. Solid or dashed lines indicate direct or indirect interactions, respectively. The various shapes represent different protein functions (see legend within **Fig. 1**).
(PDF)

Figure S3 **Western-blot analysis for the differential expression of HSP60 in the RCC cell lines.** The bottom panel represents the scanning densitometry for respective protein expression between *VHL* genotypes of RCC.
(PDF)

Figure S4 **Representative Progenesis LC-MS (Nonlinear Dynamics) analysis.** **A.** *m/z* 522.2911 (2+) with the aligned retention time of 27.0 min identified as differentially regulated; **B.** One of the sequence-identifying MS/MS (CID) spectra, precursor *m/z* 522.3: SNTPILVDGK corresponding to glucose-6-phosphate isomerase (IPI00027497) with a score of 25.17; **C.** Average normalized abundances shown here tag this protein as being down-regulated in *VHL*-mutant RCC.
(PDF)

Figure S5 **Representative Progenesis LC-MS (Nonlinear Dynamics) analysis.** **A.** *m/z* 617.3656 (2+) with the aligned retention time of 44.2 min identified as differentially regulated; **B.** One of the sequence-identifying MS/MS (CID) spectra, precursor *m/z* 617.4: IFGVTTLDIVR corresponding to mitochondrial malate dehydrogenase 2 (IPI00291006) with a score of 45.68; **C.** Average normalized abundances shown here tag this protein as being up-regulated in *VHL*-mutant RCC.
(PDF)

Table S1 **Differentially expressed proteins of** ***VHL*** **-wt (Caki-2) and** ***VHL*** **-mut (786-O) RCCs identified by database search (Mascot) and quantified by extracted peptide intensity (MS) features generated with Progenesis LC-MS.** Minimum confidence scores: protein 99%, peptide 95% with a minimum of 2 peptides identified (validation by Scaffold). ANOVA was accepted at *P*<0.05 with at least a 2-fold change in protein abundance.
(DOCX)
