# Supplementary figures and images for: Proteomic Analysis of Signaling Network Regulation in Renal Cell Carcinomas with Differential Hypoxia-Inducible Factor-2α Expression
Source: PLoS One. 2013 Aug 5;8(8):e71654. doi: 10.1371/journal.pone.0071654 (PMC3733962; doi:10.1371/journal.pone.0071654)

**Figure S1**

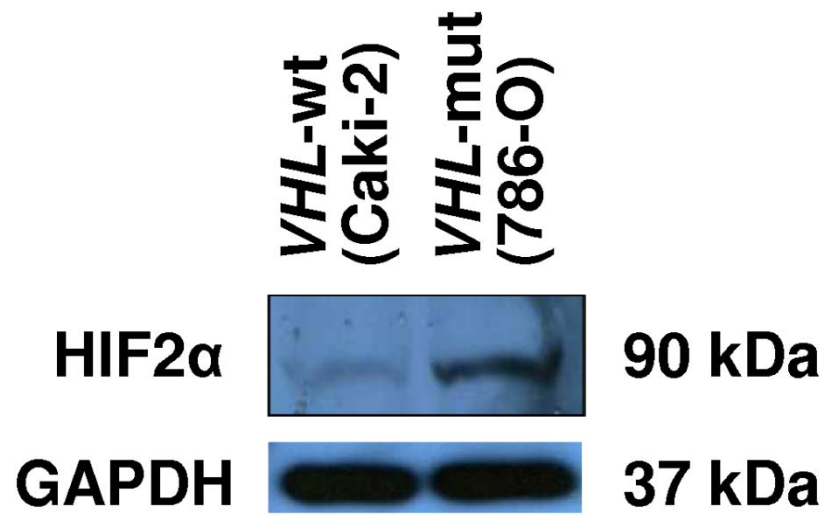

Supplement: Figure S1 — Western-blot analysis for the expression of HIF2α in 786-O ( VHL -mut) and Caki-2 ( VHL -wt) RCC cell lines. (PDF) [file pone.0071654.s001.pdf]

Figure S2

A

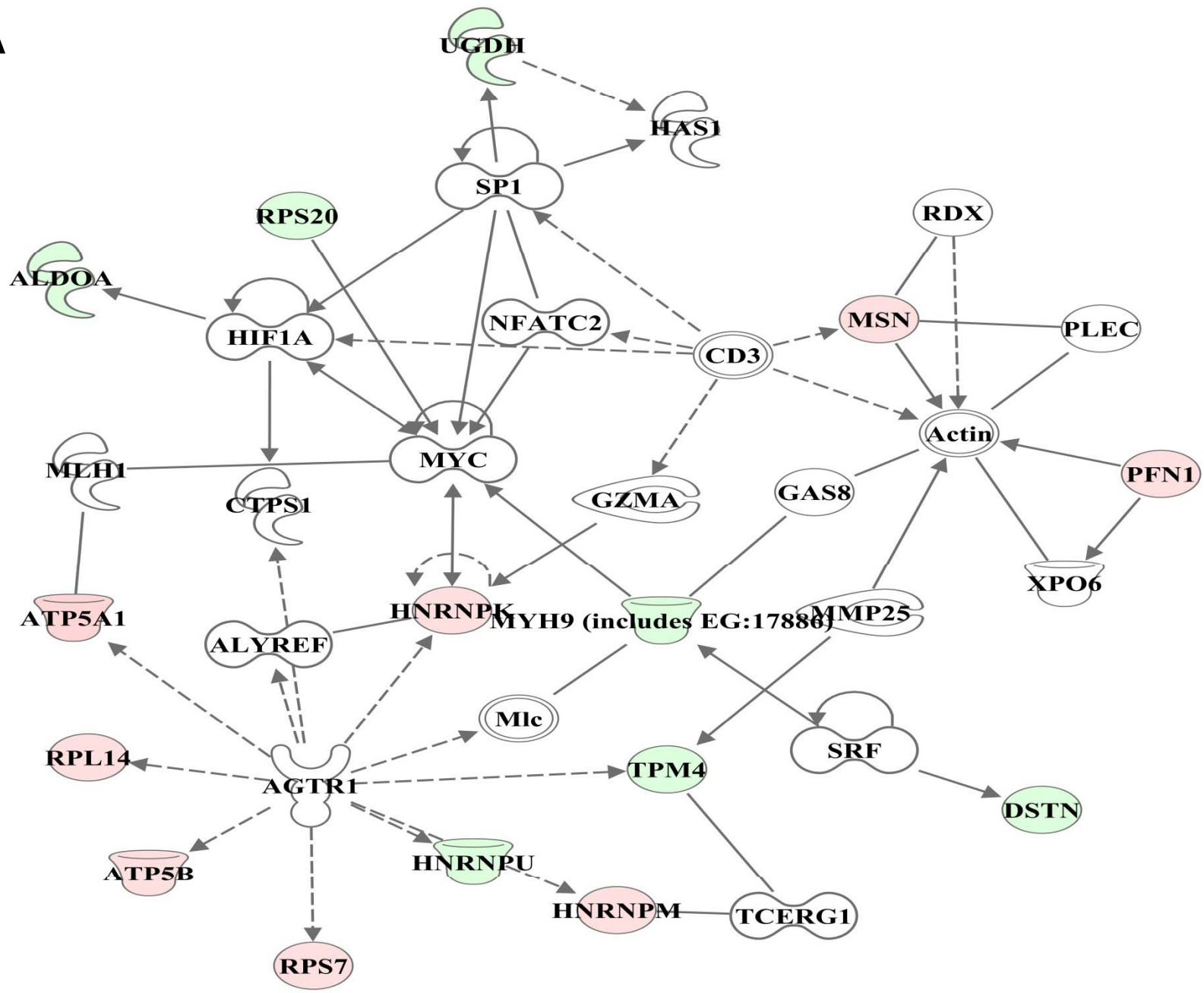

**B**

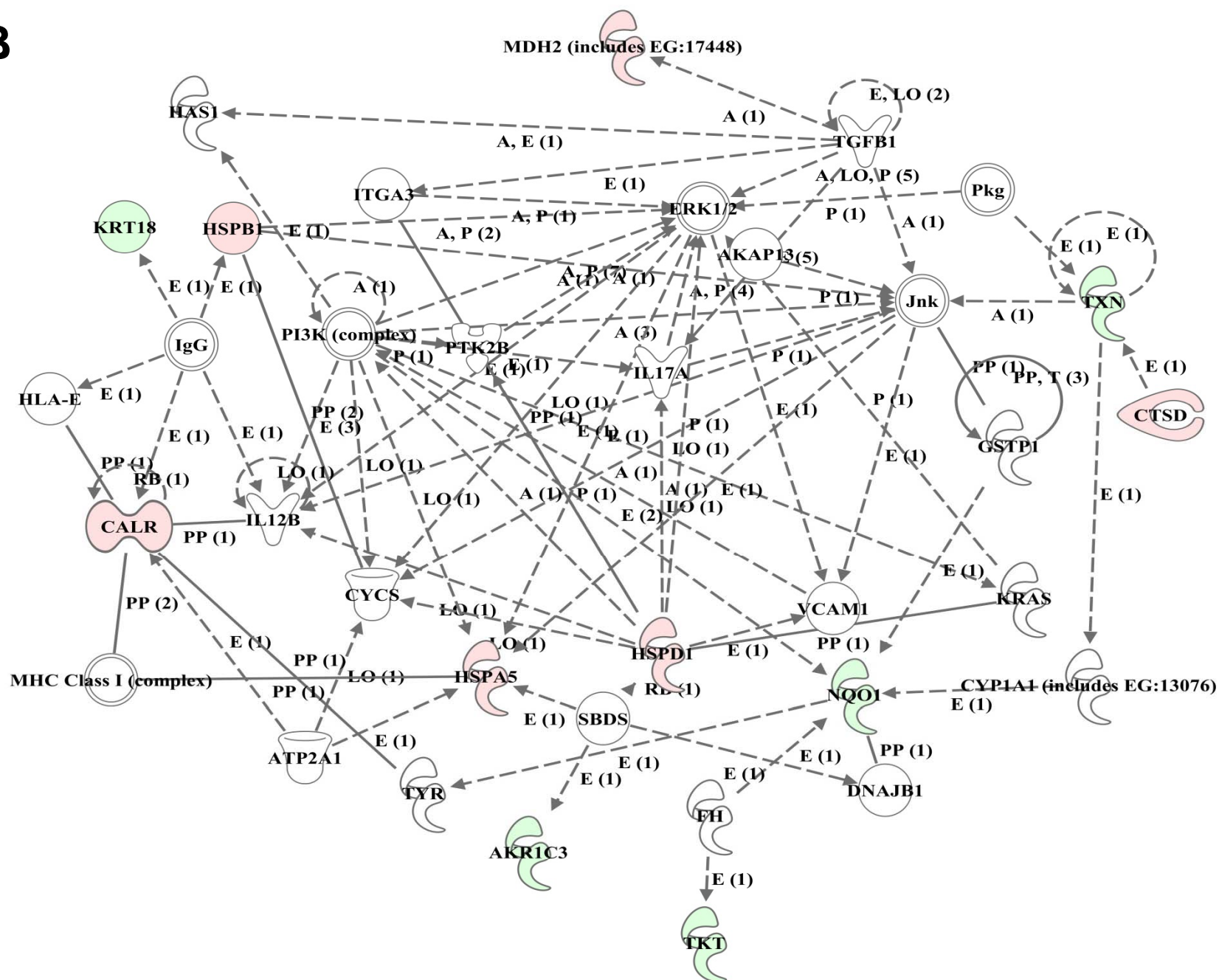

**Figure S2 (cont.)**

**C**

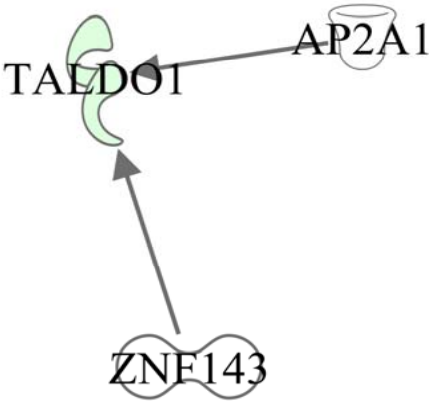

### Figure S2 (cont.)

# D

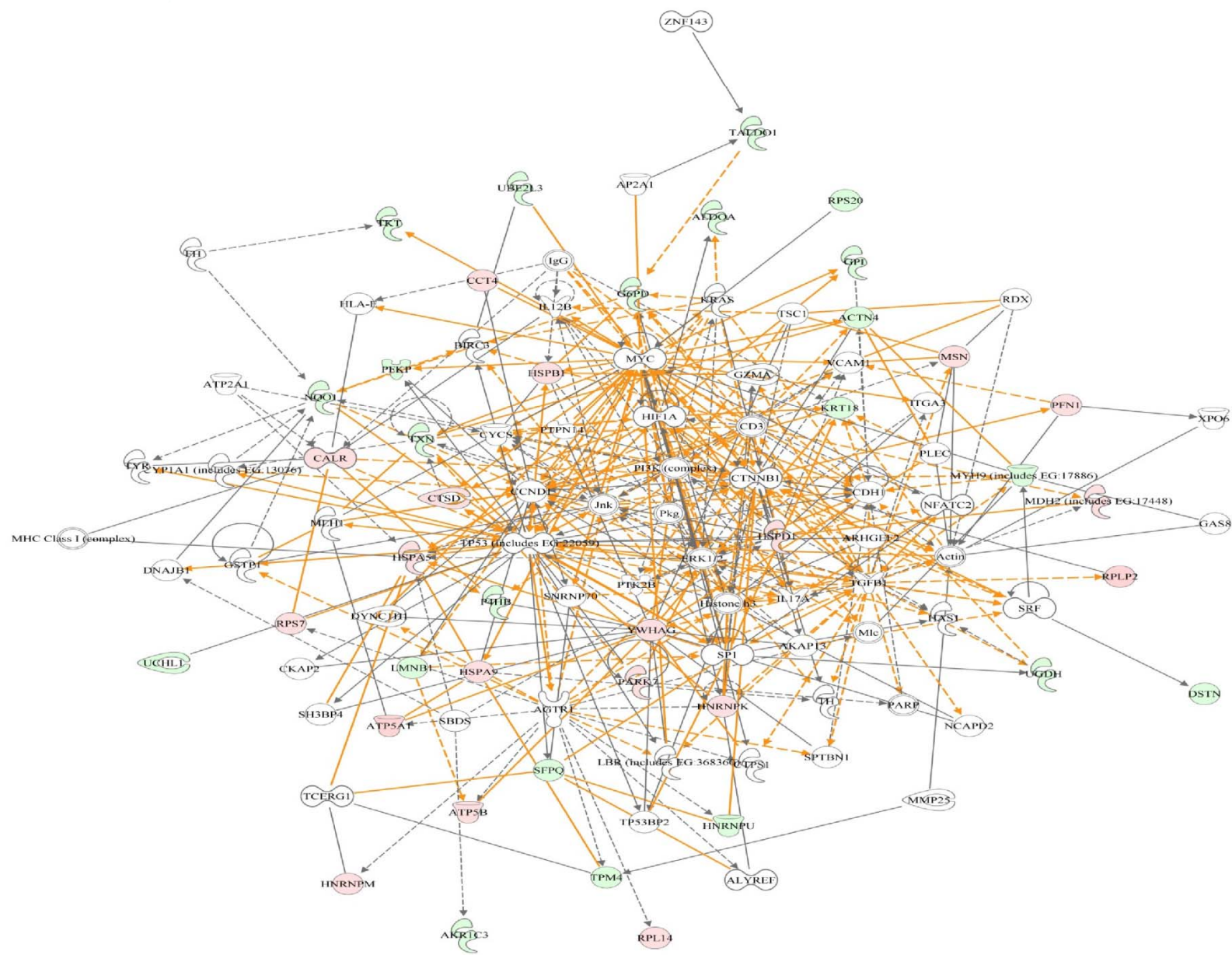

Supplement: Figure S2 — Ingenuity Pathway Analyses (IPA). Proteins with significant differential expression regulate: A. A network of nucleic acid metabolism, small molecule biochemistry, cellular assembly and organization; B. A network of cellular development, cellular growth and proliferation, hematological system development and function; C. A network of hereditary disorder, metabolic disorder, carbohydrate metabolism; D. The complex, merged network that can be built from networks shown in Fig. 1 and Fig. S2A–C. Red shading: up-regulated in VHL-mut RCC; green shading, down-regulated in VHL-mut RCC. Protein–protein interactions from the network diagram are represented by single lines and proteins/compounds that regulate another protein are indicated by arrows. Solid or dashed lines indicate direct or indirect interactions, respectively. The various shapes represent different protein functions (see legend within Fig. 1 ). (PDF) [file pone.0071654.s002.pdf]

**Figure S3**

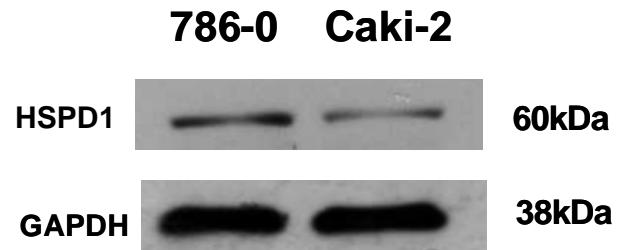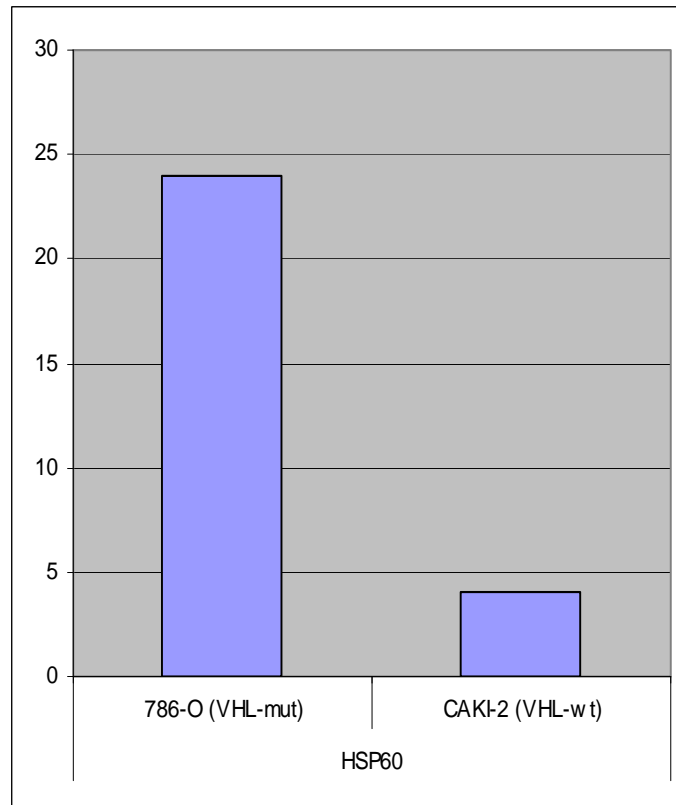

Supplement: Figure S3 — Western-blot analysis for the differential expression of HSP60 in the RCC cell lines. The bottom panel represents the scanning densitometry for respective protein expression between VHL genotypes of RCC. (PDF) [file pone.0071654.s003.pdf]

Figure S4

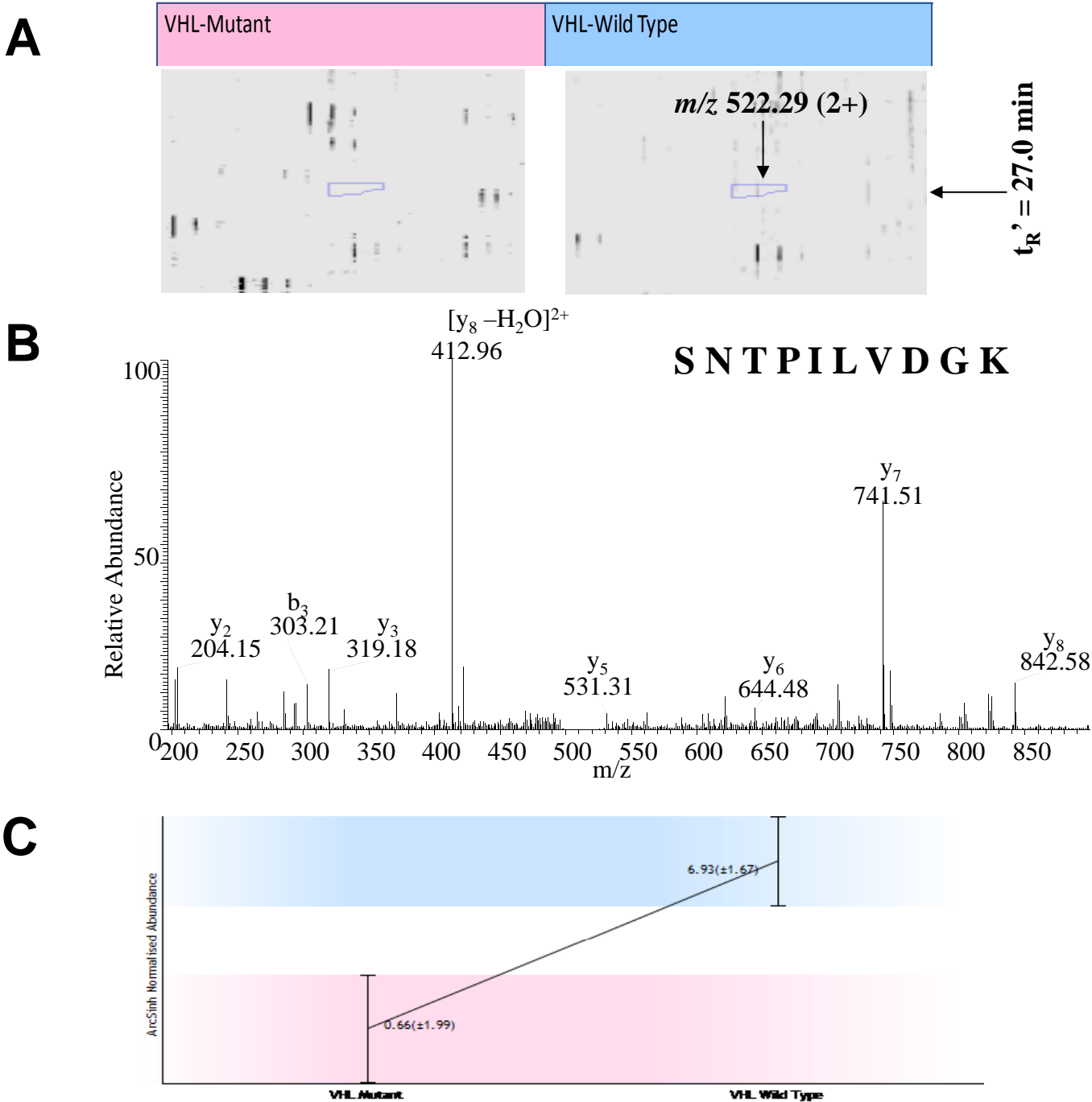

Supplement: Figure S4 — Representative Progenesis LC-MS (Nonlinear Dynamics) analysis. A. m/z 522.2911 (2+) with the aligned retention time of 27.0 min identified as differentially regulated; B. One of the sequence-identifying MS/MS (CID) spectra, precursor m/z 522.3: SNTPILVDGK corresponding to glucose-6-phosphate isomerase (IPI00027497) with a score of 25.17; C. Average normalized abundances shown here tag this protein as being down-regulated in VHL-mutant RCC. (PDF) [file pone.0071654.s004.pdf]

Figure S5

**A**

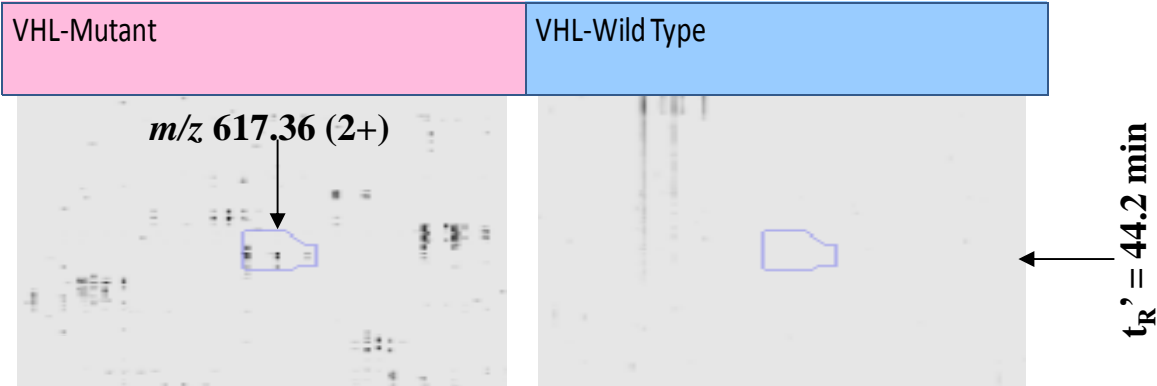

**B**

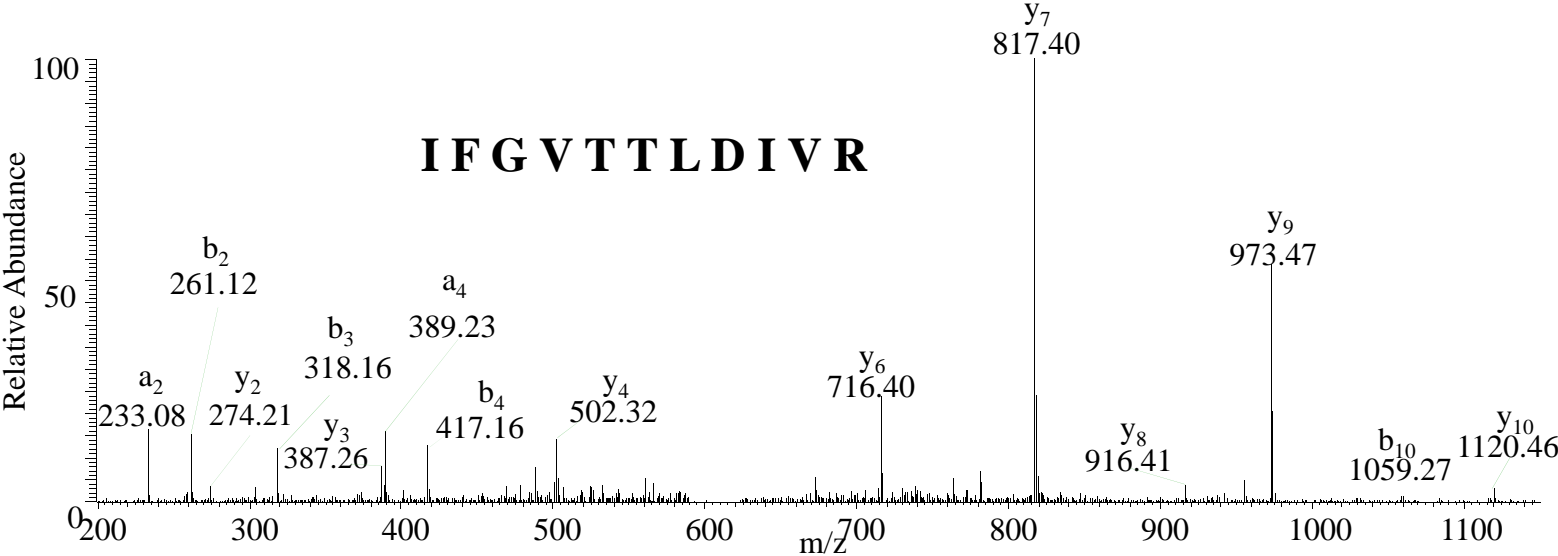

**C**

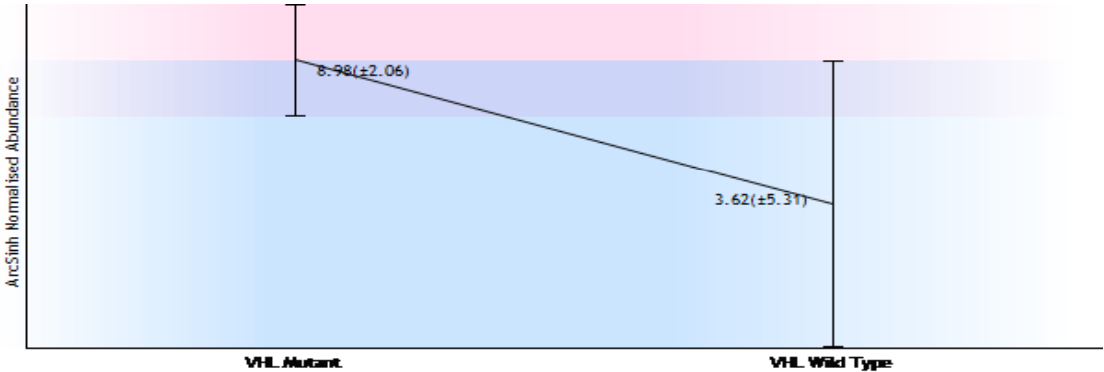

Supplement: Figure S5 — Representative Progenesis LC-MS (Nonlinear Dynamics) analysis. A. m/z 617.3656 (2+) with the aligned retention time of 44.2 min identified as differentially regulated; B. One of the sequence-identifying MS/MS (CID) spectra, precursor m/z 617.4: IFGVTTLDIVR corresponding to mitochondrial malate dehydrogenase 2 (IPI00291006) with a score of 45.68; C. Average normalized abundances shown here tag this protein as being up-regulated in VHL-mutant RCC. (PDF) [file pone.0071654.s005.pdf]
