# Supplementary material for: Proteomic Analysis of Signaling Network Regulation in Renal Cell Carcinomas with Differential Hypoxia-Inducible Factor-2α Expression
Source: PLoS One. 2013 Aug 5;8(8):e71654. doi: 10.1371/journal.pone.0071654 (PMC3733962; doi:10.1371/journal.pone.0071654)
Supplement: Table S1 — Differentially expressed proteins of VHL -wt (Caki-2) and VHL -mut (786-O) RCCs identified by database search (Mascot) and quantified by extracted peptide intensity (MS) features generated with Progenesis LC-MS. Minimum confidence scores: protein 99%, peptide 95% with a minimum of 2 peptides identified (validation by Scaffold). ANOVA was accepted at P<0.05 with at least a 2-fold change in protein abundance. (DOCX) [file pone.0071654.s006.docx]

Table S1. Differentially expressed proteins of *VHL*-wt (Caki-2) and *VHL*-mut (786-O) RCCs identified by database search (Mascot) and quantified by extracted peptide intensity (MS) features generated with Progenesis LC-MS. Minimum confidence scores: protein 99%, peptide 95% with a minimum of 2 peptides identified (validation by Scaffold). ANOVA was accepted at *P* < 0.05 with at least a 2-fold change in protein abundance.

| **Accession** | | **Protein Name**^a^ | | | **Molecular Weight**  **(kDa)** | ***P* ANOVA** | | **Max Fold Change** | | ***VHL*-mutant (786-O) Average Normalized Abundance±Standard Deviation** | ***VHL*-wild type (Caki-2) Average Normalized Abundance±Standard Deviation** | **Number of Unique Peptides** | | **Sequence Coverage (%)** | | |  |  |  |  |  |  |  |  |
| --- | --- | --- | --- | --- | --- | --- | --- | --- | --- | --- | --- | --- | --- | --- | --- | --- | --- | --- | --- | --- | --- | --- | --- | --- |
| **A) Up-regulated in VHL-mutant (786-O)** | | | |  | |  | |  | |  |  |  | |  | | |  |  |  |  |  |  |  |  |
| IPI00003362 | | | 78 kDa glucose-regulated  Protein (*HSPA5*) | 72 | | 0.04 | | 3.4 | | 2.71E+05 ± 1.36E+05 | 7.85E+04 ± 4.09E+04 | 13 | | 22 | | |  |  |  |  |  |  |  |  |
| IPI00007765 | | | Stress-70 protein, mitochondrial (*HSPA9*) | 74 | | 0.03 | | 12.5 | | 2.55E+04 ± 1.09E+04 | 2.04E+03 ± 1.10E+03 | 7 | | 9 | | |  |  |  |  |  |  |  |  |
| IPI00008529 | | | 60S acidic ribosomal protein P2 (*RPLP2*) | 12 | | 0.03 | | 43.2 | | 1.21E+05 ± 9.53E+04 | 2.81E+03 ± 9.21E+02 | 4 | | 51 | | |  |  |  |  |  |  |  |  |
| IPI00011229 | | | Cathepsin D (*CTSD*) | 45 | | 0.01 | | 2.4 | | 8.96E+04 ± 1.10E+04 | 3.77E+04 ± 1.98E+04 | 5 | | 11 | | |  |  |  |  |  |  |  |  |
| IPI00013415 | | | 40S ribosomal protein S7 (*RPS7*) | 22 | | 0.01 | | 4.0 | | 2.94E+04 ± 7.27E+03 | 7.28E+03 ± 3.82E+03 | 4 | | 32 | | |  |  |  |  |  |  |  |  |
| IPI00020599 | | | Calreticulin (*CALR*) | 48 | | 0.02 | | 3.3 | | 2.56E+05 ± 7.88E+04 | 7.85E+04 ± 3.76E+04 | 7 | | 25 | | |  |  |  |  |  |  |  |  |
| IPI00024915 | | | Peroxiredoxin-5, mitochondrial (*PRDX5*) | 22 | | 0.004 | | 210.0 | | 3.12E+04 ± 1.87E+04 | 1.49E+02 ± 8.87E+01 | 2 | | 16 | | |  |  |  |  |  |  |  |  |
| IPI00025512 | | | Heat shock protein beta-1 (*HSPB1*) | 23 | | 0.001 | | 11.1 | | 1.64E+06 ± 9.84E+05 | 1.47E+05 ± 6.13E+04 | 9 | | 66 | | |  |  |  |  |  |  |  |  |
| IPI00171903 | | | Isoform 1 of heterogeneous nuclear ribonucleoprotein M (*HNRNPM*) | 78 | | < 0.001 | | 3.5 | | 3.10E+05 ± 3.11E+04 | 8.75E+04 ± 1.82E+04 | 17 | | 13 | | |  |  |  |  |  |  |  |  |
| IPI00216049 | | | Isoform 1 of heterogeneous nuclear ribonucleoprotein K (*HNRNPK*) | 51 | | 0.03 | | 6.3 | | 2.51E+05 ± 1.57E+05 | 3.99E+04 ± 6.31E+03 | 13 | | 23 | | |  |  |  |  |  |  |  |  |
| IPI00216691 | | | Profilin-1 (*PFN1*) | 15 | | 0.005 | | 11.4 | | 2.09E+06 ± 1.42E+06 | 1.82E+05 ± 4.92E+04 | 9 | | 56 | | |  |  |  |  |  |  |  |  |
| IPI00219365 | | | Moesin (*MSN*) | 68 | | 0.002 | | 4.4 | | 4.29E+04 ± 5.28E+03 | 9.85E+03 ± 5.90E+03 | 18 | | 25 | | |  |  |  |  |  |  |  |  |
| IPI00220642 | | | 14-3-3 protein gamma (*YWHAG*) | 28 | | 0.006 | | 5.2 | | 1.04E+04 ± 3.70E+03 | 1.99E+03 ± 4.69E+02 | 6 | | 32 | | |  |  |  |  |  |  |  |  |
| IPI00298547 | | | Protein DJ-1 (*PARK7*) | 20 | | < 0.001 | | 16.1 | | 4.44E+04 ± 1.19E+04 | 2.75E+03 ± 7.48E+02 | 5 | | 26 | | |  |  |  |  |  |  |  |  |
| ^a^Gene symbols are given in parentheses. | | | | | | | | | | | | | | | | |  |  |  |  |  |  |  |  |
| **Table S1** (cont.) | | | | | | | | | | | | | | |  |  |  |  |  |  |  |  |  |  |
| **Accession** | | **Protein Name**^a^ | | | **Molecular Weight**  **(kDa)** | ***P* ANOVA** | | **Max Fold Change** | | ***VHL* mutant (786-O) Average Normalized Abundance±Standard Deviation** | ***VHL*-wild type (Caki-2) Average Normalized Abundance±Standard Deviation** | **Number of Unique Peptides** | | **Sequence Coverage (%)** | | |  |  |  |  |  |  |  |  |
| **A) Up-regulated in VHL-mutant (786-O)** | | | | | | | | | | | | | | | | |  | |  |  |  |  |  |  |
| IPI00302927 | | | T-complex protein 1 subunit delta (*CCT4*) | 58 | | 0.04 | | 5.1 | | 2.48E+04 ± 7.62E+03 | 4.88E+03 ± 1.86E+03 | 6 | | 8 | | |  |  |  |  |  |  |  |  |
| IPI00453473 | | | Histone H4 (*HIST1H4C;HIST2H4B;HIST1H4A;HIST1H4L;HIST1H4J;HIST1H4E;HIST1H4D;HIST1H4I;HIST1H4F;HIST1H4B;HIST4H4;HIST2H4A;HIST1H4K;HIST1H4H*) | 11 | | 0.009 | | 5.8 | | 4.76E+04 ± 1.27E+04 | 8.18E+03 ± 3.17E+03 | 5 | | 34 | | |  |  |  |  |  |  |  |  |
| IPI00465028 | | | Triosephosphate isomerase isoform 2 (*TPI1P1*;*TPI1*) | 31 | | 0.03 | | 3.2 | | 1.42E+06 ± 6.53E+05 | 4.46E+05 ± 1.97E+05 | 16 | | 61 | | |  |  |  |  |  |  |  |  |
| IPI00555744 | | | Ribosomal protein L14 variant (*RPL14*) | 24 | | 0.008 | | 29.5 | | 2.35E+04 ± 1.84E+04 | 7.96E+02 ± 7.94E+02 | 5 | | 15 | | |  |  |  |  |  |  |  |  |
| **B) Down-regulated in VHL-mutant (786-O)** | | | |  | |  | |  |  |  | |  | |  | | |  |  |  |  |  |  |  |  |
| IPI00009790 | | 6-phosphofructokinase type C (*PFKP*) | | 86 | | 0.03 | | 3.0 | 3.06E+04 ± 6.28E+03 | 9.29E+04 ± 2.32E+04 | | 17 | 24 | |  |  |  |  |  |  |  |  |  |  |
| IPI00010740 | | Long isoform of proline and glutamine-rich splicing factor (*SFPQ*) | | 76 | | 0.002 | | 5.5 | 5.02E+03 ± 1.68E+03 | 2.76E+04 ± 6.84E+03 | | 6 | 10 | |  |  |  |  |  |  |  |  |  |  |
| IPI00010779 | | Isoform 1 of tropomyosin alpha-4 chain (*TPM4*) | | 29 | | 0.02 | | 22.5 | 1.39E+03 ± 7.85E+02 | 3.13E+04 ± 1.74E+04 | | 5 | 38 | |  |  |  |  |  |  |  |  |  |  |
| IPI00010796 | | Protein disulfide-isomerase (*P4HB*) | | 57 | | 0.04 | | 10.2 | 6.27E+03 ± 1.82E+03 | 6.39E+04 ± 4.22E+04 | | 13 | 19 | |  |  |  |  |  |  |  |  |  |  |
| IPI00012069 | | NAD(P)H dehydrogenase [quinone] 1 (*NQO1*) | | 31 | | 0.008 | | 9.8 | 4.53E+03 ± 1.16E+03 | 4.44E+04 ± 2.14E+04 | | 5 | 18 | |  |  |  |  |  |  |  |  |  |  |
| IPI00012493 | | 40S ribosomal protein S20 (*SNORD54;RPS20*) | | 13 | | 0.04 | | 7.3 | 4.16E+03 ± 3.28E+03 | 3.04E+04 ± 1.93E+04 | | 3 | 23 | |  |  |  |  |  |  |  |  |  |  |
| ^a^Gene symbols are given in parentheses. | | | | | | | | | | | | | | |  |  |  |  |  |  |  |  |  |  |
| **Table S1** (cont.) | | | | | | | | | | | | | | |  |  |  |  |  |  |  |  |  |  |
| **Accession** | | **Protein Name**^a^ | | | **Molecular Weight**  **(kDa)** | ***P* ANOVA** | | **Max Fold Change** | | ***VHL* mutant (786-O) Average Normalized Abundance±Standard Deviation** | ***VHL*-wild type (Caki-2) Average Normalized Abundance±Standard Deviation** | **Number of Unique Peptides** | | **Sequence Coverage (%)** | | |  |  |  |  |  |  |  |  |
| **B) Down-regulated in VHL-mutant (786-O)** | | | | | | | | | | | | | | |  |  |  |  |  |  |  |  |  |  |
| IPI00013808 | | Alpha-actinin-4 (*ACTN4*) | | 105 | | 0.03 | | 3.7 | 4.57E+04 ± 2.13E+04 | 1.69E+05 ± 6.72E+04 | | 32 | 37 | |  |  |  |  |  |  |  |  |  |  |
| IPI00018352 | | Ubiquitin carboxyl-terminal hydrolase isozyme L1 (*UCHL1*) | | 25 | | 0.04 | | 10.4 | 1.45E+03 ± 9.44E+02 | 1.51E+04 ± 7.82E+03 | | 8 | 44 | |  |  |  |  |  |  |  |  |  |  |
| IPI00019502 | | Isoform 1 of myosin-9 (*MYH9*) | | 227 | | 0.02 | | 3.2 | 5.19E+04 ± 2.61E+04 | 1.66E+05 ± 4.83E+04 | | 75 | 36 | |  |  |  |  |  |  |  |  |  |  |
| IPI00021347 | | Ubiquitin-conjugating enzyme E2 L3 (*UBE2L3*) | | 18 | | 0.003 | | 3.1 | 3.19E+02 ± 3.19E+02 | 9.90E+02 ± 3.59E+02 | | 3 | 24 | |  |  |  |  |  |  |  |  |  |  |
| IPI00105407 | | Aldo-keto reductase family 1 member B10 (*AKR1B10*) | | 36 | | < 0.001 | | 17.1 | 1.82E+04 ± 7.02E+03 | 3.11E+05 ± 8.75E+04 | | 14 | 47 | |  |  |  |  |  |  |  |  |  |  |
| IPI00216008 | | Glucose-6-phosphate 1-dehydrogenase (*G6PD*) | | 64 | | < 0.001 | | 8.3 | 3.77E+03 ± 1.63E+03 | 3.14E+04 ± 1.29E+04 | | 5 | 5 | |  |  |  |  |  |  |  |  |  |  |
| IPI00216298 | | Thioredoxin (*TXN*) | | 12 | | 0.03 | | 2.6 | 1.49E+04 ± 5.82E+03 | 3.92E+04 ± 9.25E+03 | | 5 | 40 | |  |  |  |  |  |  |  |  |  |  |
| IPI00216730 | | Histone H2A type 2-B (*HIST2H2AB*) | | 14 | | 0.01 | | 229.3 | 4.68E+01 ± 4.22E+01 | 1.07E+04 ± 5.71E+03 | | 8 | 55 | |  |  |  |  |  |  |  |  |  |  |
| IPI00217975 | | Lamin-B1 (*LMNB1*) | | 66 | | 0.004 | | 5.8 | 3.83E+03 ± 9.39E+02 | 2.22E+04 ± 5.32E+03 | | 8 | 7 | |  |  |  |  |  |  |  |  |  |  |
| IPI00291483 | | Aldo-keto reductase family 1 member C3 (*AKR1C3*) | | 37 | | 0.006 | | 30.5 | 1.60E+03 ± 7.83E+02 | 4.88E+04 ± 9.55E+03 | | 3 | 35 | |  |  |  |  |  |  |  |  |  |  |
| IPI00465439 | | Fructose-bisphosphate aldolase A (*ALDOA*) | | 39 | | 0.005 | | 6.1 | 1.46E+05 ± 2.24E+04 | 8.81E+05 ± 3.19E+05 | | 23 | 44 | |  |  |  |  |  |  |  |  |  |  |
| IPI00473014 | | Destrin (*DSTN*) | | 19 | | < 0.001 | | 19.9 | 3.60E+03 ± 1.50E+03 | 7.15E+04 ± 1.64E+04 | | 3 | 22 | |  |  |  |  |  |  |  |  |  |  |
| IPI00479217 | | Short isoform of heterogeneous nuclear ribonucleoprotein U (*HNRNPU*) | | 89 | | 0.04 | | 2.5 | 2.62E+04 ± 1.29E+04 | 6.54E+04 ± 1.96E+04 | | 8 | 9 | |  |  |  |  |  |  |  |  |  |  |
| IPI00554788 | | Keratin, type I cytoskeletal 18 (*KRT18*) | | 48 | | 0.01 | | 2.6 | 6.75E+04 ± 2.37E+04 | 1.74E+05 ± 3.55E+04 | | 16 | 33 | |  |  |  |  |  |  |  |  |  |  |
| IPI00643920 | | cDNA FLJ54957, highly similar to transketolase (*TKT*) | | 69 | | 0.01 | | 4.0 | 6.24E+04 ± 2.50E+04 | 2.48E+05 ± 9.43E+04 | | 20 | 24 | |  |  |  |  |  |  |  |  |  |  |
| IPI00744692 | | Transaldolase (*TALDO1*) | | 38 | | 0.02 | | 20.8 | 6.35E+02 ± 2.00E+02 | 1.32E+04 ± 5.62E+03 | | 12 | 32 | |  |  |  |  |  |  |  |  |  |  |

^a^Gene symbols are given in parentheses.
